# A single-beat algorithm to discriminate farfield from nearfield bipolar voltage electrograms from the pulmonary veins

**DOI:** 10.1007/s10840-023-01535-7

**Published:** 2023-04-04

**Authors:** Vincent Schlageter, Patrick Badertscher, Adrian Luca, Philipp Krisai, Florian Spies, Thomas Kueffer, Stefan Osswald, Jean-Marc Vesin, Michael Kühne, Christian Sticherling, Sven Knecht

**Affiliations:** 1 Department of Cardiology, University Hospital Basel, University of Basel, Basel, Switzerland; 2 Cardiovascular Research Institute Basel, University Hospital Basel, University of Basel, Basel, Switzerland; 3 Department of Cardiology, Lausanne University Hospital, Lausanne, Switzerland; 4 Department of Cardiology, Inselspital, Bern University Hospital, University of Bern, Bern, Switzerland; 5 Applied Signal Processing Group, Swiss Federal Institute of Technology, Lausanne, Switzerland

**Keywords:** Nearfield, Farfield, Pulmonary vein isolation, Machine learning, Bipolar voltage electrogram

## Abstract

**Background:**

Superimposition of farfield (FF) and nearfield (NF) bipolar voltage electrograms (BVE) complicates the confirmation of pulmonary vein (PV) isolation after catheter ablation of atrial fibrillation. Our aim was to develop an automatic algorithm based on a single-beat analysis to discriminate PV NF from atrial FF BVE from a circular mapping catheter during the cryoballoon PV isolation.

**Methods:**

During freezing cycles in cryoablation PVI, local NF and distant FF signals were recorded, identified and labelled. BVEs were classified using four different machine learning algorithms based on four frequency domain (high-frequency power (P_HF_), low-frequency power (P_LF_), relative high power band, P_HF_ ratio of neighbouring electrodes) and two time domain features (amplitude (V_max_), slew rate). The algorithm-based classification was compared to the true identification gained during the PVI and to a classification by cardiac electrophysiologists.

**Results:**

We included 335 BVEs from 57 consecutive patients. Using a single feature, P_HF_ with a cut-off at 150 Hz showed the best overall accuracy for classification (79.4%). By combining P_HF_ with V_max_, overall accuracy was improved to 82.7% with a specificity of 89% and a sensitivity of 77%. The overall accuracy was highest for the right inferior PV (96.6%) and lowest for the left superior PV (76.9%). The algorithm showed comparable accuracy to the classification by the EP specialists.

**Conclusions:**

An automated farfield-nearfield discrimination based on two simple features from a single-beat BVE is feasible with a high specificity and comparable accuracy to the assessment by experienced cardiac electrophysiologists.

**Graphical Abstract:**

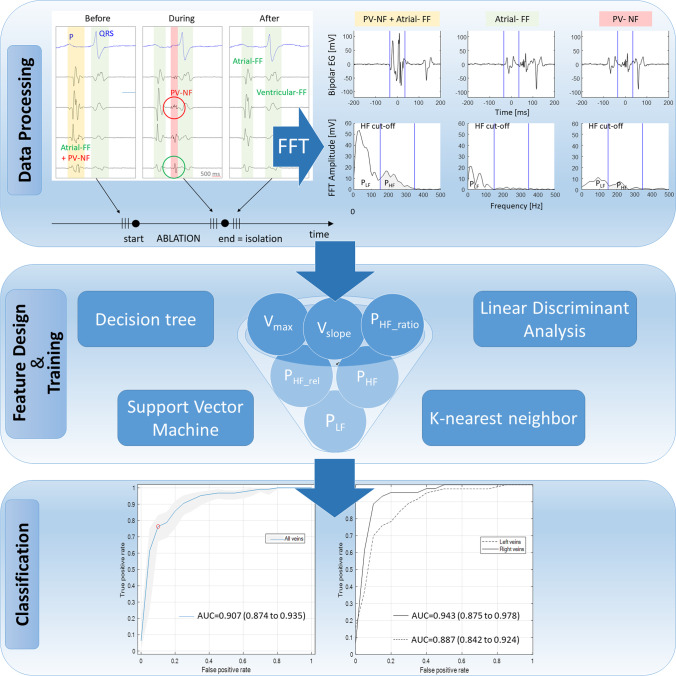

**Supplementary Information:**

The online version contains supplementary material available at 10.1007/s10840-023-01535-7.

## Introduction

Pulmonary vein (PV) isolation (PVI) is the cornerstone of catheter ablation for the treatment of atrial fibrillation [1]. A lesion set around the PV antrum is performed using point-by-point radiofrequency or “single-shot” ablation devices. Without PV ectopy, the endpoint of the intervention is the electrical elimination of the local PV electrogram using a circular mapping catheter (CMC) placed distal to the ablation lesion (entrance block). The bipolar voltage electrogram (BVE) measured between two electrodes of the CMC may, however, not only consist of nearfield (NF) local PV BVEs representing the target for ablation, but also be superimposed by farfield (FF) atrial BVE arising from the surrounding structures such as the right atrium, the left atrial appendage or the left atrium (LA). Discrimination of FF from NF BVE can be challenging, especially when both BVE temporally overlap. By pacing manoeuvres from the distal coronary sinus or the left atrial appendage, discrimination between FF and NF BVE can be facilitated [2, 3]. However, especially for single-shot devices such as cryoballoon (CB) catheters, additional diagnostic catheters enabling such pacing manoeuvres are often not advanced into the LA.

Temporal splitting of the CMC BVE into FF and NF with subsequent disappearance of the FF BVE is an established endpoint of the freezing cycle of CB PVI. However, due to the design-specific distance of the CMC from the occluded ostium during the freezing cycle, PV BVE are not always present at the location of the CMC during the freeze. Furthermore, the CMC sometimes needs to be positioned even deeper in the PV for stabilization purpose without penetrating myocardial sleeves. For this condition, ostial confirmation before and after ablation needs to be performed without the benefit of visible signal splitting and disappearance. To address this challenge of FF vs NF discrimination, a single-beat algorithm independent of the ablation technique would be beneficial to identify PV NF to assess the success of an ablation. Such an algorithm would allow as well for a reliable confirmation of PVI using the CMC during RF PVI, since only one CMC is commonly used and synchronous disappearing of the PV BVE in the other ipsilateral vein cannot be documented.

The aim of the current study was to develop an automatic algorithm based on a single-beat analysis to discriminate PV nearfield from LA farfield BVE from a circular mapping catheter after cryoballoon PVI.

## Method

### Specifications

We included consecutive patients referred for first-time catheter ablation of atrial fibrillation in which a cryoballoon system (Arctic Front Advanced, Medtronic, USA) in combination with an inner lumen diagnostic CMC catheter (Achieve, Medtronic) was used. All patients signed informed consent prior to the study, which was approved by the local ethics committee (Ethics Committee Northwest and Central Switzerland) and conducted in accordance with the Declaration of Helsinki. The authors had full access to and take full responsibility for the integrity of the data.

The loop diameter of the octapolar CMC was 20 mm with an inter-electrode spacing of 10 mm and an electrode size of 1mm. Only patients in sinus rhythm during ablation enabling them to identify a distinct PVI with BVE from the PVs showing delay and subsequent disappearance during the freezing cycle (representing entrance block) were included in the study. Intracardiac bipolar signals were acquired and stored using a standard electrophysiology system (Sensis, Siemens Healthineers, Erlangen, Germany). For intracardiac signals, a high-pass and low-pass filter with cut-offs at 30 Hz and 300 Hz respectively was used. A 50-Hz notch filter was enabled for all signals, which were recorded with a sampling rate of 2000 Hz.

### Cryoballoon ablation

Cryoballoon ablation was performed under conscious sedation as published in detail elsewhere [4]. Briefly after a single transseptal puncture, the CB in combination with the CMC catheter was advanced into the LA through a steerable sheath (FlexCath Advance, Medtronic). With the CMC positioned in the PV distal to the CB, the CB was inflated and advanced to the ostium of the PV. Confirmation of PV occlusion with contrast injection was performed at the physician’s discretion. Before the freezing cycle was started, the CMC was retracted to the CB catheter tip to enable PV BVE documentation. The start of the freeze and the instance of complete PV isolation were tagged in the EP system for subsequent BVE characterization.

### Manual electrogram classification

**Fig. 1:**
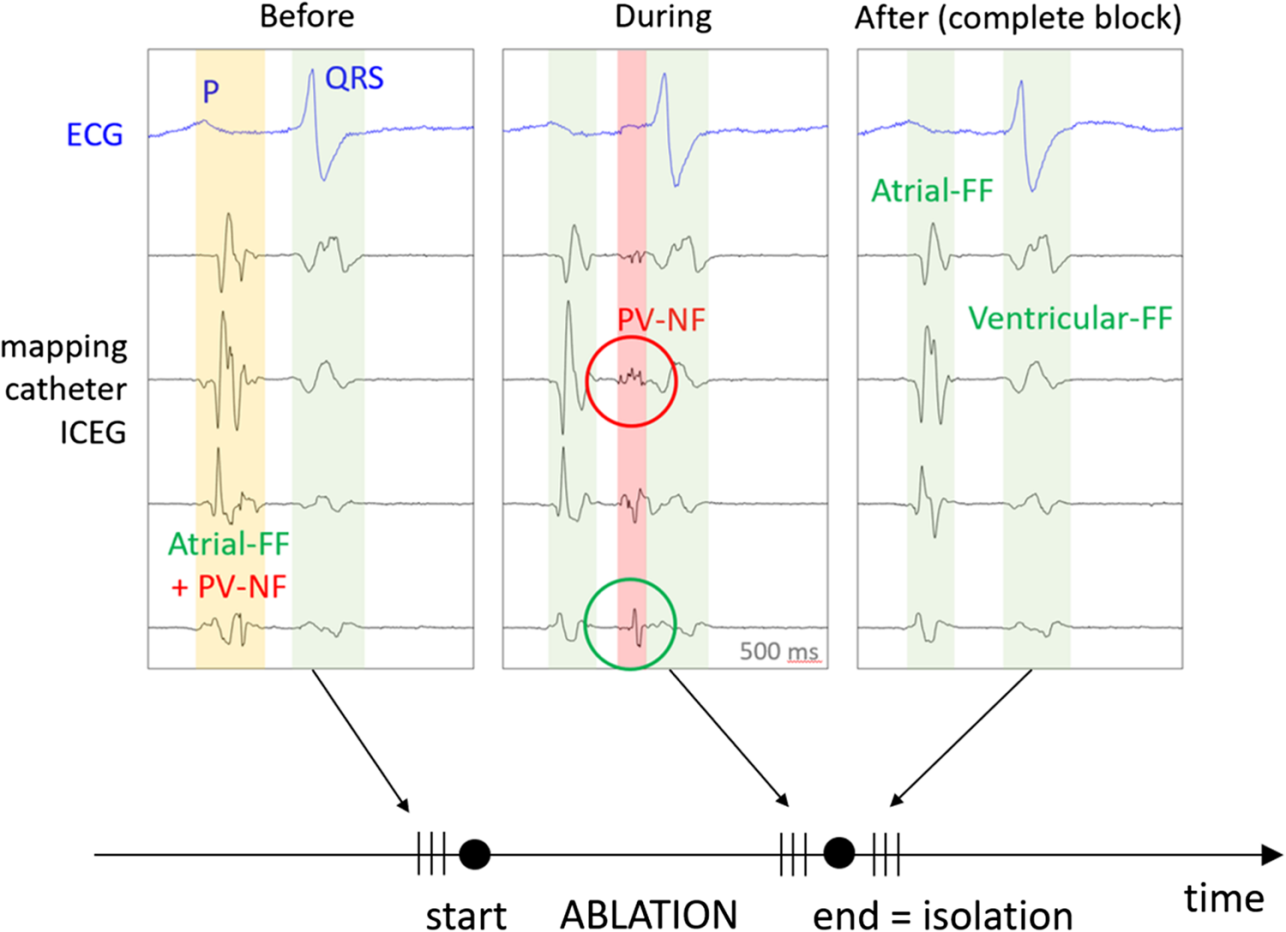
Representative bipolar voltage electrograms (BVE). The width of the coloured block represents the onset and offset of the analysed electrogram. Before ablation (left column): BVE within a yellow block was defined as combined FF-NF since no isoelectric line between the amplitudes could be observed. The information of a combined BVE was retrospectively gained based on the observation of the delayed PV-NF (red block) BVE during ablation (middle column). Green-circled BVE represents the FF of the red-encircled PV-NF. After isolation, only atrial-FF was visible. Ventricular-FF was excluded from the analysis. FF, farfield; ICEG, intracardiac electrogram; NF, nearfield; PV, pulmonary vein

To create the database for the development of the automatic algorithm, only freezing cycles with apparent delay and subsequent disappearance of local pulmonary vein signals, proving successful acute PVI confirmed by the operating physician and EP engineer, were included. The relevant intracardiac BVEs were imported into a custom software for manual classification by the EP engineer. With a sweep speed of 100 mm/s, the relevant BVE were reviewed from the beginning to the end of the freezing cycle and the electrograms were selected from their onset to the offset (Fig. 1). In the three beats before acute PVI, the remaining electrogram components were manually classified as atrial farfield (atrial-FF) and the disappearing electrogram as PV nearfield (PV-NF). One of the three selected beats of each group was randomly selected for further analysis. The BVEs at the start of the ablation were either classified as atrial-FF, as PV-NF or as combined atrial-FF and PV-NF (combined FF-NF) according to the observation during isolation.

### Feature development

**Fig. 2:**
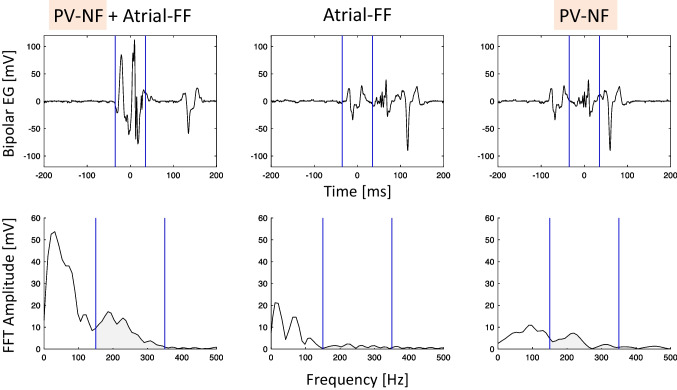
Representation of time and frequency domain measures. Time and corresponding frequency domain representation of the three exemplary classes during ablation in the same vein. The blue lines in the bipolar electrogram (EG) (upper row) represent the manually defined onset and offset of the analysed bipolar voltage electrogram. When a local PV-NF is present, high frequencies are visible in the frequency domain, as illustrated in the lower row by the highlighted 150 to 300 Hz frequency band. The area under the curve reflects the power. FF, farfield; FFT, fast Fourier transform; NF, nearfield; PV, pulmonary vein

To reduce artefacts, noisy electrograms were discarded based on the assessment of a reference period before the P-wave. Six features were extracted from the BVE to feed the classification algorithms. For the four frequency domain features, the power spectrum was calculated using the fast Fourier transform (FFT) with a window width of 35 ms and zero-padding in order to have a frequency resolution of 10 Hz. We split this spectrum into a low (P_LF_: 0 to 150 Hz) and high power frequency band (P_HF_: 150 to 300 Hz) at a cut-off of 150 Hz based on the observed characteristic of the power spectrum (Fig. 2). The power spectrum was calculated for all eight bipolar electrode pairs of the CMC while sliding the window from the onset to the offset of the BVE as delimited in the electrogram characterization step. Out of the eight electrode pairs, only the BVE having the highest absolute value of P_HF_, suggesting the closest NF source to this electrode pair, was retained. Figure 2 shows the representation in the time and frequency domains for a typical example of the three classes of BVE (PV-NF, atrial -FF, combined FF-NF). As a third frequency domain feature, we calculated the relative high power band (P_HF_rel_) as P_HF_ divided by the overall power between 0 and 300 Hz.

The ratio of the high power band (P_HF_) with the two neighbouring bipolar electrode pairs (P_HF_Neighbor_) was calculated, and the highest power ratio (P_HF_/P_HF_Neighbor_) was used as the fourth frequency domain feature. This was designed based on the hypothesis that an electrical potential generated by a close-by source should have a higher spatial resolution than a distant one [5]. To take into account the wide range of BVE amplitude, which can be explained by the number of myocardial fibre bundles, their orientation and the distance with regard to the bipolar electrode pairs, we also included the amplitude of the BVE (V_max_) as the fifth feature. Finally, we calculated the first derivative over the width of the BVEs and characterized these samples based on their proportion with a steep slew rate >0.15 V/s (slew-rate) as the sixth feature. The features were normalized to approximate Gaussian distributions in the case of skewed distribution.

Due to the relationship of the BVE characteristics with the distance of the electrical dipole source, we additionally measured the minimal 3D distance (CartoSeg, Biosense Webster, USA) of the left atrial appendage from the left superior PV on the reconstructed LA anatomy using the pre-procedural cardiac magnetic resonance imaging as an anatomical feature.

### Machine learning electrogram classification

To discriminate a FF from NF BVE based on the features extracted from the three classes (PV-NF, atrial-FF, combined FF-NF), we used the following machine learning (ML) classifiers: decision tree, linear discriminant analysis, support vector machine (SVM) and k-nearest neighbour (KNN). To protect against overfitting, we evaluated the predictive model with a 4-fold cross-validation and kept 25% of the patients as a test subset for the final estimation of the performance of the model. To calculate confidence intervals for the accuracy and ROC curves, we used bootstrapping. Feature selection was achieved by systematic experimentation, using a forward-wrapped method based on the overall accuracy of the prediction model.

### Clinical classification

To assess the clinical value of the automatic algorithm, we randomly extracted a set of 80 BVE from the overall dataset for classification by four experienced physicians and one EP engineer. The classification was performed in a single-beat time window based on the presence or absence of an NF BVE. The accuracy of their judgment was compared to the automatic results of our algorithm. Interrater reliability was assessed using the intra-class correlation coefficient (ICC).

### Statistical analysis

Continuous variables are presented as mean ± standard deviation or as median and interquartile range as appropriate. Receiver-operating characteristic (ROC) curves were generated, and the area under the curve (AUC) was calculated for uni- and multivariate analyses. For statistical analyses, we used Matlab (Mathworks, Inc., USA).

## Results

### Dataset

We analysed 57 patients for a total number of 2680 BVE (eight electrode pairs of the CMC per acquisition). The analysis was performed for the electrode pair with the highest high-frequency band power of each separate recording, resulting in 335 analysed BVEs. The examples were balanced between the two classes with the presence of PV-NF (51.3%) (Table 1). The median duration of the BVE from onset to offset was 58 ms (95% CI: 26 to 86) for PV-NF, 70 ms (95% CI: 50 to 100) for Atrial-FF and 94 ms (95% CI: 71 to 139) for combined potentials.

**Table 1:** Classification of PV signals. Dataset from 57 patients from all four PVs. The table shows the number of examples for each of the four PVs, taken before ablation, during the ablation and after the complete block. In the latter case, PV-NF is always absent, by definition. The total number of signals with a farfield component is balanced compared to signals without a farfield component (163 to 172)

Classification		LSPV	LIPV	RSPV	RIPV	Total	
Atrial-FF	Before	2	3	6	0	11	Signals without nearfield component: **163** (48.7%)
	During	32	28	15	10	85	
	After	31	21	7	8	67	
PV-NF	Before	2	3	9	0	14	Signals with nearfield component: **172** (51.3%)
	During	19	21	21	8	69	
	After						
Combined PV-NF and atrial-FF	Before	38	25	8	9	80	
	During	6	1	1	1	9	
	After						
Total		130	102	67	36	335	

### Features

The absolute high-frequency power (P_HF_) was identified as the best single-feature classification with an overall accuracy of 79.4% (Supplemental Fig. 1). Performance of other single-feature classifications can be found in Supplemental Table 1. No significant difference in variability of the features derived from the BVEs during PVI with those before and after PVI was identified. Furthermore, the feature variations over the three beats were small compared to the range of values from all BVEs, suggestive of stable feature characteristics.

### Machine learning classification

**Fig. 3:**
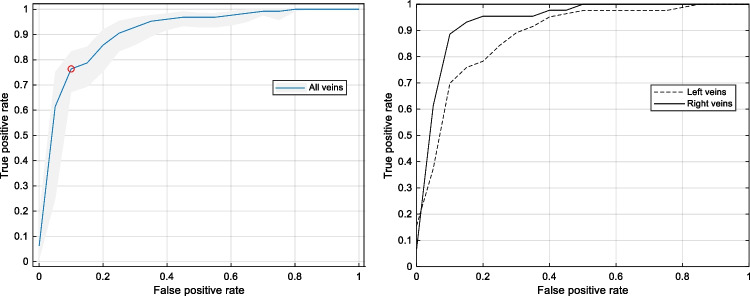
ROC curves of the classification algorithm. Left: ROC curve of the automatic classification for all veins. The red dot corresponds to the best overall accuracy. The light blue zone represents the 95% CI. AUC=0.907 (0.874 to 0.935). Right: ROC curve of the automatic classification stratified for the right PVs (plain line), AUC=0.943 (0.875 to 0.978), or left PVs (dashed line), AUC=0.887 (0.842 to 0.924).

The six frequency and time domain features were fed to the four classification algorithms. The SVM algorithm performed best and achieved a good accuracy of 82.7% (95% CI: 80.3% to 85.1%) using two features only: the power in the high-frequency band (P_HF_) and the maximal amplitude of the bipolar voltage (V_max_) (Table 2). The ROC curves of this model for all veins and the stratified right and left PVs are shown in Fig. 3. Analysing the accuracy on an individual, the PV-selective level showed an accuracy of 96.6% for the RIPV, 85.2% for the RSPV, 80.8% for the LIPV and 76.9% for the LSPV. Including the distance between LAA and LSPV as measured on the pre-procedural cardiac magnetic resonance imaging with a cut-off of < 5mm (*n*=29) or > 10mm (*n*=9) for the LSPV results in a specificity of 75% (22 of 29) and 100% (9 of 9) for the atrial-FF prediction. Adding more features to the SVM algorithm resulted in an increase of overall accuracy of only 1 or 2% at the cost of more complexity and a lower accuracy with the holdout set being suggestive of overfitting.

**Table 2:** Confusion matrix using the optimized SVM model for the classification of signals into nearfield and farfield for all veins. The SVM algorithm classified the signals correctly in 277 of 335 (82.7%) cases

True class	Nearfield *N*=172	132 (77%)	40 (23%)
	Farfield *N*=163	18 (11%)	145 (89%)
		Nearfield	Farfield
		**Predicted class**

### Clinical evaluation

The mean accuracy, sensitivity and specificity for the signal assessment by the five EP specialists were 85.2%, 91.9% and 78.5%, respectively, with an ICC of 0.69. For the same samples, the ML algorithm showed a comparable accuracy of 82.7% with a lower sensitivity (76.3%) but higher specificity (89.2%).

## Discussion

Reliable discrimination of FF from NF BVE from the pulmonary veins during catheter ablation of AF is of high clinical importance. Especially for single-shot devices such as the CB catheter, correct electrogram interpretation can be challenging. The main findings of our study are as follows: [1] For the discrimination of farfield from nearfield signals using a single feature, the absolute power in high-frequency showed the best overall accuracy with 79.4%, followed by the proportion with a slew rate > 0.15 V/s (slew-rate), voltage amplitude, absolute power in low-frequencies (P_LF_), relative high-frequency power (P_HF-rel_) and P_HF_Neighbor_. [2] With multiple features in the prediction model, the combination of a frequency domain (P_HF_) and time domain analysis (V_max_) feature yielded the best overall accuracy of 82.7% to predict NF-PV signal from a single-beat BVE. ##With the (relatively) high specificity of 89% and a sensitivity of 77%, the implementation as a diagnostic test to identify if additional ablation for PVI is needed is reasonable. [3] On a vein-selective analysis, the overall accuracy was highest for the right inferior PV (96.6%) and lowest for the left superior PV (76.9%). Implementing the information on the distance between LSPV and the left atrial appendage, the accuracy of the algorithm could be improved for the LSPV. [4] The algorithm showed numerically comparable accuracy to the classification by five experienced EP specialists. However, further external testing on larger datasets is required to confirm the results.

Intracardiac uni- and bipolar voltage electrograms are the fundamental basis for any invasive electrophysiological study. The BVE measured between two electrodes of an EP catheter is used to characterize the underlying propagation of the depolarization of the myocardial cells based on temporal activation, electrogram morphology and amplitude. Despite reflecting more local myocardium depolarization than for unipolar voltage electrogram, the BVE is still influenced to some extent by distant or farfield depolarization of the myocardial tissue. In addition to the underlying tissue characteristics, the shape and size of the EGM [6] are strongly influenced by the electrode size, inter-electrode distance and relative orientation of the bipolar electrodes in relation to the propagating wavefront of the cellular depolarization [7].

### Characteristics and features of local bipolar voltage electrogram

PV potentials are defined and colloquially described as “sharp” nearfield BVEs following a farfield BVE from the LA. Dependent on the position of the CMC within the PV, the PV signal is more or less easily discernible and separable from the atrial-FF BVE and varies between the veins [8]. This “sharpness” of the local PV BVE is mentioned throughout the publications, but to the best of our knowledge, a reproducible, quantitative measure has never been published. The sharpness of an electrogram can be defined in the time domain based on the signal width and the slew rate of the deflection. For the sensing of atrial signals in cardiovascular implantable electronic devices, for instance, an interpolated slew rate in the range of 0.5V/s is recommended as a characteristic for nearfield atrial signal detection [9]. In our study, with a “sharpness criteria” of a cumulative threshold of a slew rate above 0.15V/s for the local BVEs, this feature, however, was not identified as a reliable predictor to identify nearfield BVE. However, the frequency domain–based high power frequency spectrum at a cut-off of 150 Hz might address this lack of definition for nearfield characterization.

### Farfield elimination

Farfield (interference) elimination was aimed at using a novel dipole source model to describe the impact of the nearfield and farfield source on the measured voltage signal [10, 11]. With this approach, an improvement in the spatial resolution of the electrogram from approximately 10 to 2.5 mm was expected. The assumed spatial resolution of 10 mm with the standard voltage calculation is in line with our observation on the impact of the LAA on the LSPV BVE. With the inclusion of the distance between LSPV and LAA, we observed that our frequency-dependent algorithm works highly reliably at distances between LAA and LSPV above 10 mm with 0% false positive LA NF detection. However, with distances below 5 mm, the farfield BVE of the LAA shows similar frequency characteristics (especially the high-frequency power spectrum) with the nearfield PV signal. Using this dipole density modelling approach on our contact-based BVE might help to further improve the accuracy of our algorithm, especially for the LSPV with the LAA in close proximity to the CMC.

Another established approach to eliminate the impact of farfield on the BVE is to use dedicated catheter designs. In general, catheters with closely spaced electrodes are recommended. Computational simulations showed that smaller electrodes with narrow spacing produce sharper BVE with higher electrogram amplitude [5, 7]. However, when the option of selecting a specific catheter design is not available (as for CB PVI), the above-described frequency analysis with a dedicated high-frequency cut-off for the power spectrum might be a powerful alternative to eliminate the farfield impact of the local BVE.

### Strategies to discriminate farfield and nearfield electrograms for PVI confirmation

A simple way to discriminate between FF and NF BVE is to observe the temporal evolution of the signal during ablation. When a local PV-NF is detectable, this signal shows a temporal delay with the advancement of the lesion, allowing the definition of a local PV signal based on the progression of the PV entrance block. However, no observable delay must not inevitably imply that the vein is already isolated, since BVE might still be hidden in the LA farfield component. Refraining from further ablation, the endpoint of PVI will not be reached. On the other side, with an already isolated vein, additional unnecessary ablation might result in complications, such as phrenic nerve palsy reported for CB PVI of the RSPV.

Numerous pacing strategies have been established in clinical practice, including decremental pacing, differential pacing, perivenous pacing or intravenous pacing [3]. However, when pacing from the distal CS, overlapping of the two BVEs (PV-NF and atrial-FF) was still observed in 65% of the patients.

Another approach to differentiate PV nearfield from LA farfield has been described using a multi-electrode mapping catheter in combination with an automated software implemented in the mapping system (Rhythmia, Boston Scientific, USA) [12]. With this software (Lumipoint), areas with a simultaneous electrical activation were highlighted, allowing for an identification of a farfield effect from a surrounding structure, such as for instance the LAA. However, this approach requires a detailed electroanatomical mapping after ablation. Time domain bipolar voltage electrogram characteristics were used to define a library of characteristic PV electrograms [13]. Besides the typology (including the amplitude and the number of peaks), the minimal and maximal slope of the BVE, its peak angle and amplitude were used to characterize the BVE. Using this library, a 2-step algorithm showing an accuracy of 93% was developed. In a subsequent study, the library-dependent classification algorithm on BVE was expanded and validated to the herein-used octapolar CMC for CB PVI [14]. In contrast to this strategy, our algorithm is not dependent on a training library and has the potential to be implemented as fully automatic approach, since only two simple features are required. A frequency-based analysis of the PV BVE from a decapolar CMC with 15 mm, 20 mm or 25 mm diameter (Lasso, Biosense Webster) was already performed 10 years ago [15]. After FFT, the bimodal amplitude spectrum (similar to that shown by our study in Fig. 2) was characterized by the full-width half maximum (FWHM) for the first and maximum peak in the spectrum, similar to the low-frequency power feature in our manuscript. Furthermore, the frequency of the maximum second peak times the amplitude divided by the peak amplitude and the frequency cut-off that divides the FFT area in two equal halves were computed, similar to our frequency cut-off. Our model showed a comparable accuracy with the inclusion of only two features, making the interpretation and automatic implementation easier.

## Outlook

The herein presented algorithm for discrimination of FF from NV BVE based on frequency analysis of BVE has the potential to be applied accordingly to any catheter types. This approach might be also helpful for ablation catheters to identify the nearfield and farfield component of the BVE. With that information and the estimated lesion size, the efficacy of an ablation might be estimated. Furthermore, a catheter-specific filter applied to the BVE might allow for a local NF-based onset annotation for activation mapping.

## Limitations

Despite having analysed 335 BVEs, this is still a relatively small single-centre study investigating the feasibility of this approach. Second, the features were selected based on the balanced accuracy from the ROC curves to optimize sensitivity and specificity. For the final clinical application as a diagnostic test, a cut-off based on the improved specificity might be advisable. Third, the current algorithm is not implemented yet as a real-time algorithm and requires offline data processing. Fourth, the clinical validation and comparison with the automatic algorithm were performed by an internal dataset only, which was used as well for training. Fifth, the results are only valid for the tested catheter and need to be verified for different catheter designs. Finally, the training was performed only with BVE during sinus rhythm. If and how the algorithm will work for patients in AF needs further clarification.

## Conclusions

In conclusion, we presented and validated an automatic classification based on only two simple features extracted from a single-beat PV electrogram with a high specificity, allowing using it as a diagnostic test. A simple frequency domain analysis with a high-pass cut-off of 150 Hz seems to be reasonable for this catheter design to discriminate farfield from nearfield electrograms. The classification results were comparable to the assessment by five experienced EP specialists showing its clinical practicality.

### Supplementary information

ESM 1

## Data Availability

The data that support the findings of this study are available on request from the corresponding author.

## Abbreviations

BVE: Bipolar voltage electrogram
CMC: Circular mapping catheter
NF: Nearfield
FF: Farfield
LA: Left atrium
PV: Pulmonary vein
PVI: Pulmonary vein isolation
PV-NF: Pulmonary vein nearfield
atrial-FF: Atrial farfield
