# Supplementary material for: A single-beat algorithm to discriminate farfield from nearfield bipolar voltage electrograms from the pulmonary veins
Source: J Interv Card Electrophysiol. 2023 Apr 4;66(9):2047–54. doi: 10.1007/s10840-023-01535-7 (PMC10694100; doi:10.1007/s10840-023-01535-7)
Supplement: Supplementary file 1 — ESM 1 [file 10840_2023_1535_MOESM1_ESM.docx]

**Supplemental Material**

Supplemental Figure 1. ROC curves to compare the optimized SVM model (black line), with classification based on a single feature: High frequency band (HF) (blue line) or amplitude (Amp) (pink line). SVM – support vector machine.

Supplemental Figure 2. Exemplary bipolar voltage electrograms where both, the machine learning (ML) algorithm and the EP specialists, performed a wrong classification:

ML and EP specialists identified “nearfield” for a farfield signal:

| 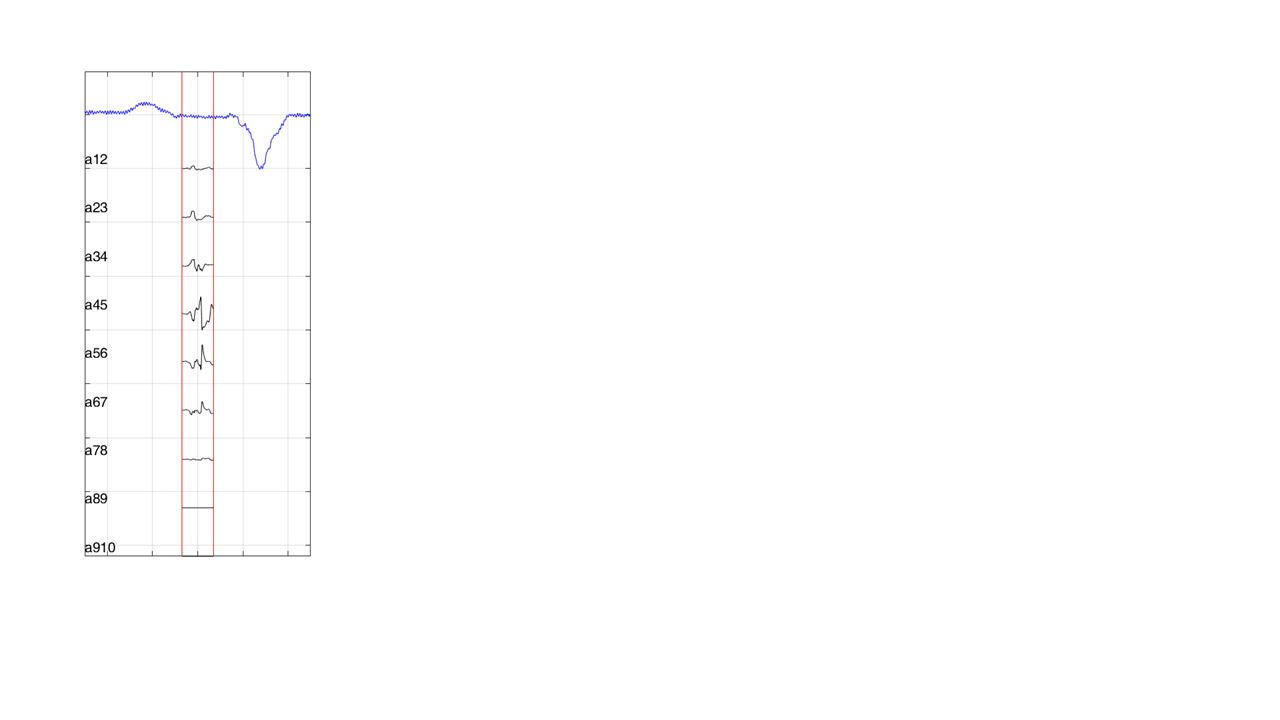 | 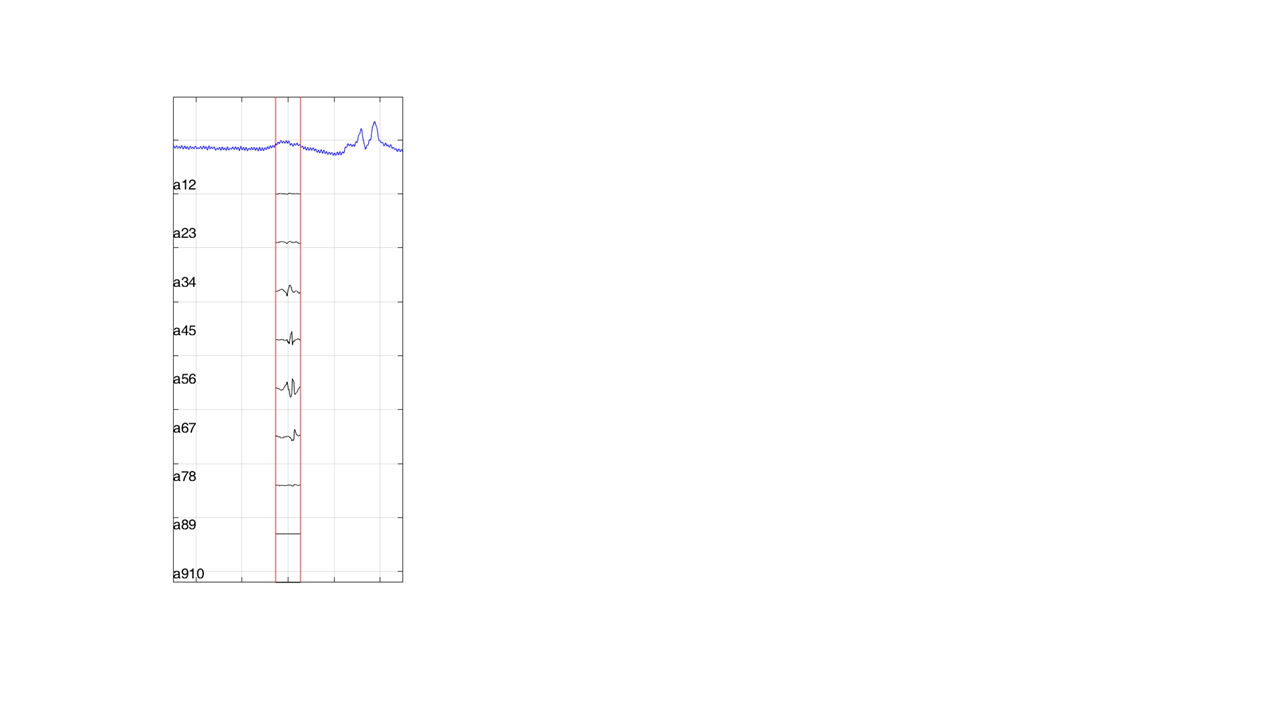 | 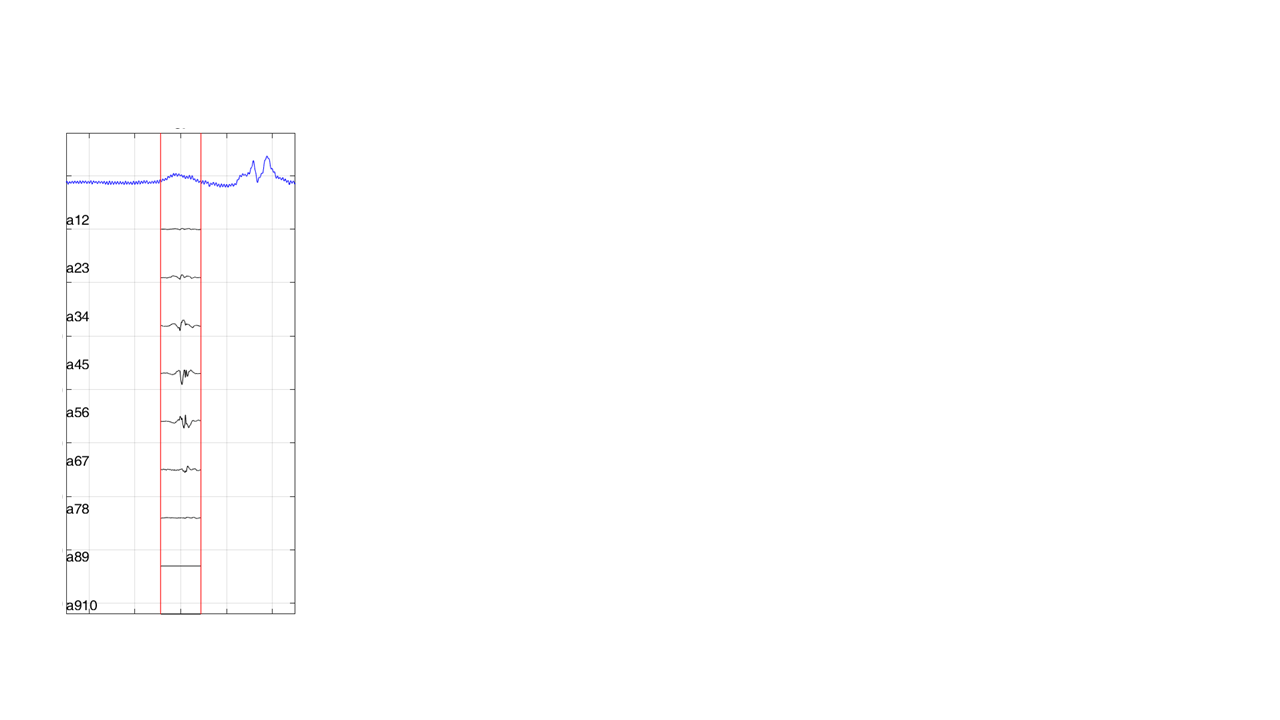 |
| --- | --- | --- |

Supplemental Figure 3. Exemplary bipolar voltage electrograms where both, the machine learning (ML) algorithm and the EP specialists, performed a wrong classification:

ML and physicians identified “farfield” of a nearfield signal:

| 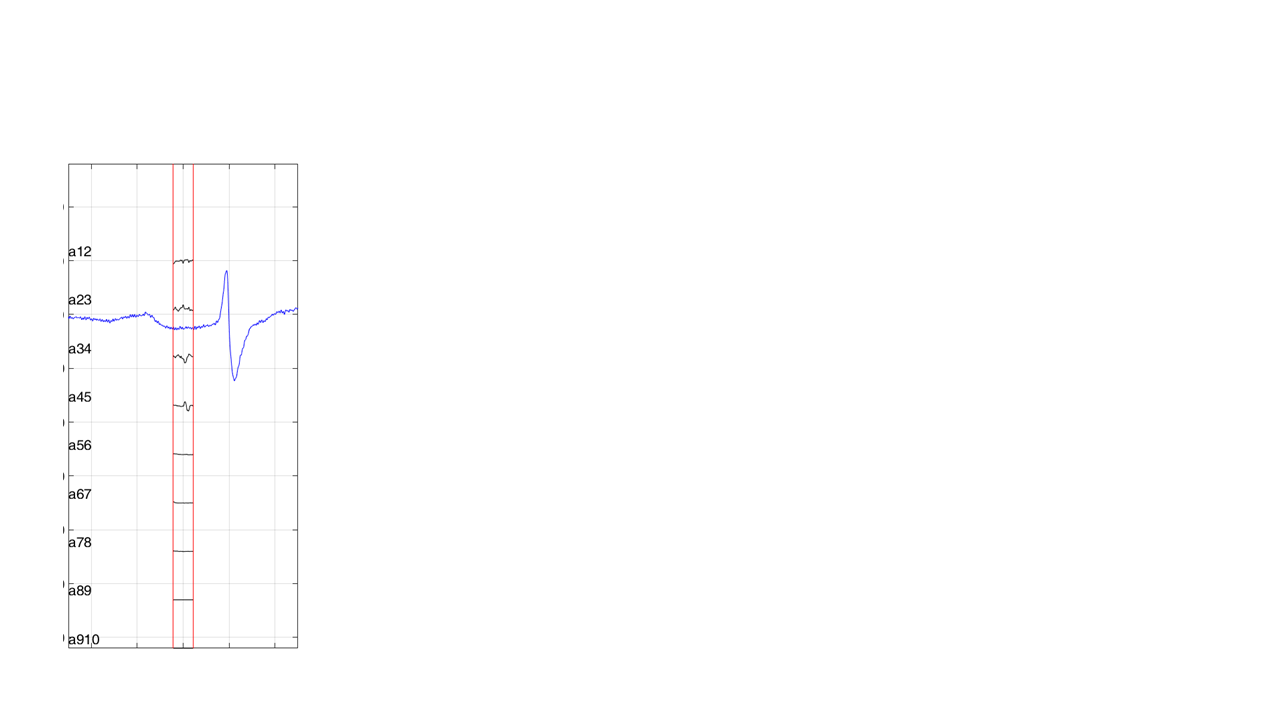 | 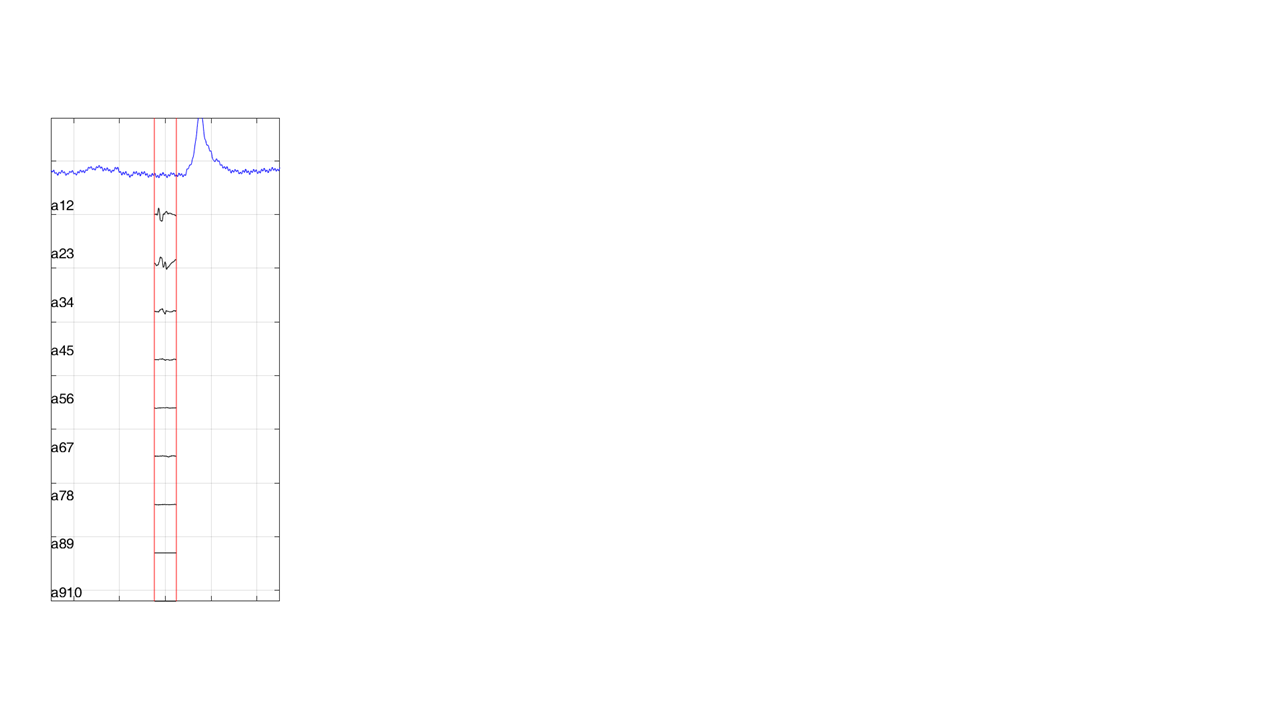 |
| --- | --- |

|  | Prediction method | Sensitivity | Specificity | Balanced Accuracy |
| --- | --- | --- | --- | --- |
| Single feature | P_HF_ | 78.7% | 80.1% | 79.4% |
|  | slew-rate | 84.0% | 64.7% | 74.4% |
|  | V_max_ | 63.0% | 85.0% | 74.0% |
|  | P_LF_ | 53.7% | 87.1% | 70.4% |
|  | P_HF-rel_ | 71.6% | 67.9% | 69.8% |
|  | P_HF_Neighbor_ | 54.2% | 82.2% | 68.2% |

Supplemental table 1: Single feature sensitivity, specificity, and balanced accuracy.

P_HF_ - high-frequency power, P_LF_ - low-frequency power, P_HF-rel_ - relative high power band, P_HF_Neighbor_ - P_HF_ ratio of neighbouring electrodes
